# High-yield bio-polyols from sugarcane bagasse: process optimization, structural analysis, and application potential

**DOI:** 10.1007/s11356-026-38351-1

**Published:** 2026-10-28

**Authors:** Sobhan Mohamadi, Soleiman Abbasi, María González-Alriols, Fabio Hernández-Ramos, Rasoul Rahnemaei

**Affiliations:** 1 Department of Soil Science, Faculty of Agriculture, Tarbiat Modares University, Tehran, Iran; 2 Food Colloids and Rheology Lab., Department of Food Science and Technology, Faculty of Agriculture, Tarbiat Modares University, Tehran, Iran; 3 Biorefinery Processes Research Group (BioRP), Chemical and Environmental Engineering Department, University of the Basque Country (EHU), San Sebastian, Spain

**Keywords:** Biomass-based polyols, Liquefaction, Polyurethane, Lignocellulosic waste valorization, Renewable feedstock, Sustainable manufacturing

## Abstract

This research addresses the management of agricultural waste and the partial substitution of petrochemicals in polyurethanes (PUs) by utilizing renewable resources. The present study evaluates, both quantitatively and qualitatively, the feasibility of producing bio-polyols from sugarcane bagasse by polyethylene glycol (PEG 400) as organic solvent. These polyols can be potentially utilized as substitute for petrochemical polyols in elastic PU formulations. The effects of process conditions (temperature, catalyst content, and sugarcane bagasse: solvent ratio) on the bio-polyol yield were evaluated and optimized using response surface methodology through a Box-Behnken design. In addition, at each experimental point, the qualitative characteristics of the resulting bio-polyols, including functional groups, hydroxyl number (*I*_OH_), acid number (*A*_n_), molecular weight (*M*_W_), functionality, viscosity, and polydispersity index (PDI) were determined. The optimal conditions identified by the model determined the most effective experimental parameters as 2 g of sugarcane bagasse, 3.13% catalyst, and a reaction temperature of 161.1 °C. Under these conditions, a polyol yield of 95.6% was achieved, with a prediction error of 4.4%. For the high‑yield samples, *I*_OH_ ranged from ∼ 195 to 233 mg KOH · g⁻^1^, M_W_ from 3839.27 to 7582.06 g · mol⁻^1^, functionality between 3.12 and 4, viscosity from 187.5 to 238.4 mPa · s, and PDI between 4.25 and 7.50. These results indicate that the synthesized bio-polyols possess physicochemical characteristics comparable to those reported for polyols used in elastic polyurethane formulations, suggesting their potential suitability for such applications. Overall, the study presents innovative strategies for cost-effective laboratory-scale production of bio-polyols, offering a pathway toward broader industrial adoption.

## Introduction

Nowadays, due to increasing environmental concerns, biomass-based polyols (bio-polyols) have become a potential alternative to petrochemical polyols for PUs synthesis (Lee and Kim 2023; Tran and Lee 2023). Moreover, utilizing agricultural waste as a cost-effective resource not only adds value by transforming residues into high-value products, but also contributes to mitigating environmental challenges. To produce bio-polyol with acceptable efficiency from residual biomass, the process must be purposefully designed to align with the desired properties of the final PU products, which can be flexible or rigid. The biomass feedstock, the ratio of feedstock to solvent, and the reaction conditions (reaction temperature and catalysts) are the most important parameters of the bio-polyol synthesis (Kosmela et al. 2019). Each parameter can influence the functional properties of the bio-polyol, and, therefore, their combined effects determine the optimal conditions.

A prominent method for synthesizing bio-polyols from agricultural and forest-derived biomass is the solvothermal liquefaction technique (Park et al. 2024). The liquefaction process effectively converts biomass into liquid in the presence of an appropriate solvent and catalyst (Olszuewski et al. 2023). The choice of solvent and its respective mixing ratio with biomass are critical factors that influence the liquefaction process and the resulting bio-polyol products. Forero et al. (2022) demonstrated that the yield of products synthesized using glycerol is superior to those produced with ethanol/water and water. Furthermore, low *M*_W_ PEG has proven to be an effective organic solvent in the context of biomass liquefaction. This effectiveness is particularly notable in targeting the cellulosic fraction because of the enhanced reactivity of short chain PEG with cellulose (Olszuewski et al. 2023). The selection of the liquefaction solvent is a critical factor governing biomass conversion efficiency and the properties of the resulting bio-polyols. Among polyhydric alcohols, polyethylene glycol (PEG) has been extensively employed because it acts not only as a solvent but also as a reactive medium capable of stabilizing degradation products generated during liquefaction. In particular, low-molecular-weight PEGs exhibit lower viscosity, improved penetration into the lignocellulosic matrix, and higher reactivity toward cellulose and hemicellulose degradation products, thereby enhancing liquefaction efficiency. Olszewski et al. (2023) demonstrated that the molecular weight of PEG significantly affects cellulose liquefaction and the properties of the produced bio-polyols, with lower-molecular-weight PEGs favoring more efficient conversion and lower-viscosity products. Similar observations were reported by Lee and Lee (2016) and Kim et al. (2015), who highlighted the effectiveness of PEG 400-based solvent systems for producing polyurethane precursors from biomass liquefaction. Consequently, PEG 400 was selected in the present study as an appropriate balance between liquefaction performance, solvent reactivity, and the production of bio-polyols with physicochemical characteristics suitable for polyurethane applications.

Additionally, the liquefaction process cannot be accomplished without the presence of a catalyst. Heterogeneous catalysts, such as iron-sulfur and zeolite, are unevenly distributed on the surface of the biomass, necessitating a larger quantity for adequate effectiveness (Breunig et al. 2018; Liao et al. 2019). Conversely, various acids and bases have been utilized as homogeneous catalysts, demonstrating greater efficacy in degrading diverse biomass types (Biswas et al. 2024). Among these acids, sulphuric acid has exhibited the highest catalytic efficiency in the biomass liquefaction process for polyol production (Wang et al. 2007). This efficiency is attributed to its elevated boiling point and strong acidity, which generates a substantial amount of hydrogen ions. These hydrogen ions facilitate the hydrolysis or alcoholysis of lignocellulose at lower temperatures (Zhang et al. 2020). However, determining the optimal amount of acid is crucial during different reactions, as the prolonged increased acid levels may even reduce efficiency (Wang and Touhedi, 2020).

Notably, the temperature and reaction time can also significantly affect the degradation potential of the organic solvent and catalyst, as well as the yield of polyols (Zhang et al. 2020). Generally, the solvothermal liquefaction method employs mild conditions (80 to 200 °C) and atmospheric pressure.

Liquefaction of agricultural wastes has been successfully performed in several research works. For instance, Kim et al. (2015) reported the successful conversion of various lignocellulosic waste (i.e., corn stover, rice straw, wheat straw, corn bran, cotton stalks, rapeseed cake, and apple pomace) into bio-polyols. Hernandez-Ramos et al. (2023) used organosolv lignin from several tree species for the same purpose. According to the existing reports (Kamalabadi Farahani et al. 2025), a substantial amount of sugarcane bagasse is annually produced as agricultural waste across the globe. It is a valuable source of cellulose (approximately 32% to 45%), hemicellulose (around 20% to 32%), and lignin (about 17% to 32%), along with other extractives. Due to its high cellulose and hemicellulose contents, it is a promising raw material for the production of value-added products (Kumar et al. 2021).

Previous studies on sugarcane bagasse liquefaction have mainly focused on alkaline conversion routes and the production of rigid polyurethane foams, with limited optimization of the liquefaction process and only partial physicochemical characterization of the resulting polyols. For example, Tran et al. (2022) demonstrated the feasibility of producing rigid polyurethane foams from sugarcane bagasse-derived bio-polyols, but their work primarily addressed the polyurethane production stage and evaluated only a limited number of processing variables. In contrast, the present study systematically investigates the acid-catalyzed liquefaction of sugarcane bagasse using PEG 400 as a reactive solvent through a Box-Behnken experimental design and response surface methodology (RSM). The combined effects of temperature, catalyst concentration, and biomass-to-solvent ratio were simultaneously evaluated to maximize liquefaction yield and establish relationships between process conditions and bio-polyol quality. In addition to liquefaction yield, the resulting bio-polyols were comprehensively characterized in terms of structural changes, hydroxyl number (*I*_OH_), acid number (*A*_n_), molecular weight (*M*_W_), functionality, viscosity, and polydispersity index (PDI), enabling the assessment of their potential suitability for polyurethane applications. To the authors’ knowledge, this work represents one of the first comprehensive optimization and characterization studies of sugarcane bagasse-derived bio-polyols obtained under acid-catalyzed PEG-based liquefaction conditions, providing new insights into the influence of processing parameters on bio-polyol properties and expanding the potential utilization of this abundant agricultural residue beyond the rigid polyurethane systems predominantly reported in the literature.

## Materials and methods

### Materials

Sugarcane bagasse was provided by Deh-khoda Agriculture Company (Khuzestan, Iran). Sulphuric acid (96%) and PEG 400 (industrial grade) were purchased from Panreac (Barcelona, Spain). Acetone (analytical grade), dimethylformamide (DMF, for HPLC ≥ %99.9), lithium bromide, 1,4-dioxane (analytical grade), phthalic anhydride (98%), acetonitrile (HPLC grade), and toluene-4-sulfonyl-isocyanate (TSI) (96%) from Sigma-Aldrich, and potassium hydrogen phthalate (KHP) (99%) were supplied by Fisher Scientific (MA, USA) and used without any further purification.

### Sugarcane bagasse preparation

Sugarcane bagasse was oven-dried (42 °C for 48 h), ground, sieved (#18), and the collected powder packed in polyethylene bags and stored (− 18 °C).

### Bio-polyol synthesis

Different amounts of sugarcane bagasse powder (2, 4, and 6 g) were mixed with 25 mL of PEG (corresponding sugarcane bagasse powder: solvent ratios 1:12.5, 2:12.5, and 3:12.5, respectively) in a conical flask (250 mL). Then, sulphuric acid (1.5, 3.0, and 4.5% w/w of total weight of mixture), as a catalyst, was added, and the mixtures were heated on a magnetic heater stirrer (120, 150, and 180 °C, at 500 rpm for 2 h). To prevent volume loss (evaporation of solvent at high temperatures), the conical flask was sealed, and the solvent vapors were collected using a condenser. However, solvent recovery and reuse were not quantitatively evaluated in the scope of the present study. Upon the completion of the reaction, the samples were gradually cooled to room temperature. Then, they were diluted with acetone and subsequently filtered using cellulose paper (pore size 90 nm) to remove any unreacted solids. The acetone was then separated using vacuum evaporator (41 °C) and weighed. The retentate was also oven-dried (105 °C, 72 h) and weighed. The yield of liquefaction process was calculated using Eq. 1.

1 $$\mathrm{L}\mathrm{i}\mathrm{q}\mathrm{u}\mathrm{e}\mathrm{f}\mathrm{a}\mathrm{c}\mathrm{t}\mathrm{i}\mathrm{o}\mathrm{n}\;\mathrm{Y}\mathrm{i}\mathrm{e}\mathrm{l}\mathrm{d}\;\left(\mathrm{\%}\right)=\frac{(\mathrm{I}\mathrm{n}\mathrm{i}\mathrm{t}\mathrm{i}\mathrm{a}\mathrm{l}\;\mathrm{w}\mathrm{e}\mathrm{i}\mathrm{g}\mathrm{h}\mathrm{t}\;\mathrm{o}\mathrm{f}\;\mathrm{d}\mathrm{r}\mathrm{y}\;\mathrm{s}\mathrm{u}\mathrm{g}\mathrm{a}\mathrm{r}\mathrm{c}\mathrm{a}\mathrm{n}\mathrm{e}\;\mathrm{b}\mathrm{a}\mathrm{g}\mathrm{a}\mathrm{s}\mathrm{s}\mathrm{e}-\mathrm{w}\mathrm{e}\mathrm{i}\mathrm{g}\mathrm{h}\mathrm{t}\;\mathrm{o}\mathrm{f}\;\mathrm{r}\mathrm{e}\mathrm{t}\mathrm{e}\mathrm{n}\mathrm{t}\mathrm{a}\mathrm{t}\mathrm{e})}{\mathrm{i}\mathrm{n}\mathrm{i}\mathrm{t}\mathrm{i}\mathrm{a}\mathrm{l}\;\mathrm{w}\mathrm{e}\mathrm{i}\mathrm{g}\mathrm{h}\mathrm{t}\;\mathrm{o}\mathrm{f}\;\mathrm{d}\mathrm{r}\mathrm{y}\;\mathrm{s}\mathrm{u}\mathrm{g}\mathrm{a}\mathrm{r}\mathrm{c}\mathrm{a}\mathrm{n}\mathrm{e}\;\mathrm{b}\mathrm{a}\mathrm{g}\mathrm{a}\mathrm{s}\mathrm{s}\mathrm{e}}\times 100$$

### Experimental design and optimization of liquefaction process using RSM

To investigate the effects of temperature, sugarcane bagasse content, and catalyst content on the liquefaction of sugarcane bagasse, a Box–Behnken design (BBD) with three central points was used within a response surface methodology (RSM). The independent variables chosen were temperature (*X*_1_), sugarcane bagasse content (*X*_2_), and catalyst content (*X*_3_). Each factor in the experiment was established and coded at three different levels, low (− 1), medium (0), and high (+ 1) (Table 1). The used *X*_1_ values were 120, 150, and 180 ($^\circ{\mathrm{C}}$), those of *X*_2_ were 2, 4, and 6 (g), and those of *X*_3_ were 1.5, 3.0, and 4.5 (% w/w), respectively. A total of 15 experimental runs, including replicates at center point, were carried out. The selected responses for analysis were liquefaction yield of sugarcane bagasse, *A*_n_ (mg KOH g⁻^1^), *I*_OH_ (mg KOH g⁻^1^), *M*_w_ (g mol⁻^1^), functionality (number), viscosity (mPa · s), and PDI.

**Table 1:** Variables considered in the study

Variable	Description	Units	Nomenclature	Value
Fixed	Time	min	t	120
	Organic solvent	mL	PEG 400	25
Independent	Temperature	°C	T	120–180
	Bagasse content	g	Bagasse content	2–6
	Catalyst	(w/w)*	Acid content	1.5–4.5
Dependents	Yield	(%)	Yield	0–100

^*^w/w of total weight of mixture

To optimize the liquefaction process, RSM was applied to evaluate the influence of independent variables on studied responses. A three block Box-Behnken Design comprising 15 experimental runs, including three center points, was selected for the design and optimization. The experimental data were modeled using a second-order polynomial equation (Eq. 2):

2 $${y}_{j}={b}_{0}+ \sum\nolimits_{i=1}^{3}{b}_{i}{x}_{i}+ \sum \sum\nolimits_{i<j=2}^{3}{b}_{ij}{x}_{i}{x}_{j}+\sum\nolimits_{i=1}^{3}{b}_{ii}{x}_{i}^{2}+\varepsilon$$

where *y*_j_ represents each studied response (%). The terms *b*_0_, *b*_i_, *b*_ii_, and *b*_ij_ represent the regression coefficients estimated from the experimental data using the least-squares method. The variables *x*_i_ and *x*_j_ correspond to the dimensionless, normalized independent variables. The experimental variables considered in this study include fixed factors, independent variables, and dependent variables, along with the corresponding variation ranges are presented in Table 1.

The adequacy of the model was confirmed through the evaluation of lack of fit, the coefficient of determination (*R*^2^), the significance of the regression coefficients, and the *F*-test value obtained from the analysis of variance.

Numerical optimization utilizing the multiple response function was performed. To verify the predicted optimal concentrations of the mixture and ensure conformity with the anticipated response, experiments were conducted using the specified concentrations of variables under same experimental conditions. The experimental design, statistical analysis, and regression modelling were performed using Design-Expert (version 11.0, Stat-Ease Inc., Minneapolis, MN, USA).

### Determination of physicochemical properties of bio-polyols

The influence of liquefaction conditions on the quality and structural properties of the bio-polyols was evaluated based on the analysis of their physico-chemical characteristics. The *I*_OH_ (mg KOH g^−1^) and *A*_n_ (mg KOH g^−1^) were determined according to the ASTM E 1899-16 and ASTM D 974 (ASTM International, 2016) standards. Based on Eq. 3, samples were weighed into the titration vessel and dissolved in 10 mL acetonitrile and the solution was stirred for 30 s. Then, TSI solution (10 ml), prepared according to ASTM E 1899-16, was added; the sample was covered and stirred for 5 min. Afterwards, deionized water (0.5 ml) was added, the lid was closed again, and the mixture stirred (60 s). Finally, acetonitrile (40 ml) was added, and the mixture titrated (tetra butyl ammonium hydroxide, 0.1 M) to the second end point.

For *A*_n_, 0.4 g of bio-polyol was dissolved in 50 mL of 1,4-dioxane:water (4:1 v/v) mixture and back titrated with KOH (0.1 M in ethanol). Potentiometric titration was used to detect the equivalence point by using an automatic titrator 888 (Titrando Metrohm) and Tiamo 2.5 software. To eliminate the effect of un-reacted acid, the *A*_n_ was subtracted from the *I*_OH_ (ASTM International, 2016; Methods 2000).

3 $$\mathrm{M}\mathrm{a}\mathrm{s}\mathrm{s}\;\mathrm{o}\mathrm{f}\;\mathrm{s}\mathrm{a}\mathrm{m}\mathrm{p}\mathrm{l}\mathrm{e} \left(\mathrm{g}\right)=\frac{40}{\mathrm{E}\mathrm{x}\mathrm{p}\mathrm{e}\mathrm{c}\mathrm{t}\mathrm{e}\mathrm{d}\;\mathrm{O}\mathrm{H}\;\mathrm{v}\mathrm{a}\mathrm{l}\mathrm{u}\mathrm{e}}$$

The average *M*_W_ and the number average (*M*_n_) of bio-polyols were determined by using high-performance size exclusion chromatography (HPSEC) equipped with a Jasco LC Net II/ADC with a refractive index detector with two PolarGel-M columns (300 mm × 7.5 mm) in series. The column was kept at 40 °C, and dimethylformamide with 0.1% of lithium bromide was employed as the eluent (flow rate 0.7 mL min^−1^). Polystyrene standards (Sigma-Aldrich) (62,500–266 g mol^−1^) were used to perform the calibration curves. The functionality and PDI for bio-polyol samples were calculated using Eqs. 4 and 5:

4 $$\mathrm{F}\mathrm{u}\mathrm{n}\mathrm{c}\mathrm{t}\mathrm{i}\mathrm{o}\mathrm{n}\mathrm{a}\mathrm{l}\mathrm{i}\mathrm{t}\mathrm{y}= \frac{{\mathrm{M}}_{\mathrm{n}}\bullet {\mathrm{I}}_{\mathrm{O}\mathrm{H}}}{56100}$$

5 $$PDI=\frac{{M}_{w}}{{M}_{n}}$$

where the *M*_n_ represents the total number of molecules in a unit mass of polymer and OH index (*I*_OH_).

The apparent viscosity of bio-polyols was measured using a Fungilab viscometer equipped with a low-viscosity adapter (cylindrical spindles TL2 or TL3) at a constant rotational speed (200 rpm).

### Structural analysis of bio-polyols

Structural changes and functional groups of bagasse and bio-polyols were analyzed through ATR-FTIR analysis using a PerkinElmer, Spectrum Two spectrometer equipped with a universal attenuated total reflectance (ATR) accessory featuring an internal reflection diamond crystal. A total of 64 scans in transmission mode were collected at a resolution of 4 cm^−1^ over a broad wavenumber range (4000 to 600 cm^−1^). FT-IR spectra were recorded and compared.

## Results and discussion

### Optimization of the liquefaction conditions

**Fig. 1:**
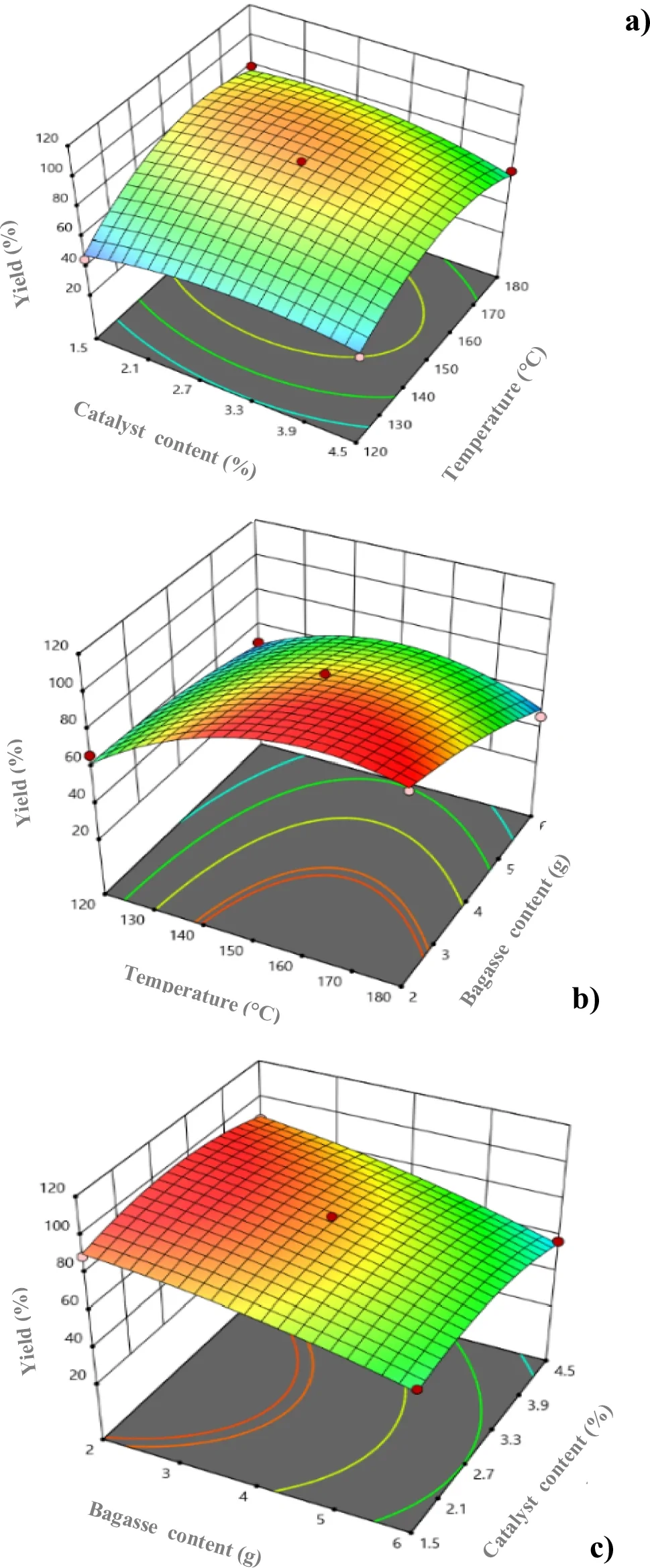
Three-dimensional plots of **a** temperature vs catalyst content, **b** temperature vs bagasse content, and **c** bagasse vs catalyst content on the yield of bio-polyol

The 15-run experimental and corresponding responses are presented in Table 2 and Fig. 1. ANOVA analysis (Table 3) revealed that only the yield model showed adequate fit (*p* < 0.0001, *R*^2^ = 0.92). No adequate models were fitted for other responses (*A*_n_, *I*_OH_, *M*_W_, functionality, viscosity, PDI; all *p* > 0.05, *R*^2^ < 0.70).

**Table 2:** Effect of temperature, bagasse, and catalyst content on sugarcane bagasse liquefaction. Bold entries indicate the samples with the highest values for the yield

Sample’s number	*T*	Bagasse	Catalyst	Yield	*A*_n_	*I*_OH_	MW*	Functionality	Viscosity	PDI**
	(°C)	(g)	(% w/w)	(%)	(mg KOH g^−1^)	(mg KOH g^−1^)	(g mol^−1^)	number	(mPa · s)	
1	150	6.00	4.50	57.43	23.13	182.40	22,926.39	4.20	469.03	18.40
2	150	4.00	3.00	87.34	14.35	239.12	5934.76	4.10	227.06	6.20
3	180	2.00	3.00	94.91	18.00	223.76	7582.06	4.03	197.93	7.50
4	150	2.00	4.50	88.21	24.41	232.96	4414.97	4.00	187.56	4.60
5	150	4.00	3.00	90.66	15.40	218.15	17,470.76	4.27	297.06	15.90
6	150	4.00	3.00	84.47	16.26	203.58	9758.73	3.53	305.40	10.00
7	120	4.00	4.50	50.57	27.11	227.50	2380.82	3.50	241.43	2.75
8	120	2.00	3.00	67.01	16.76	214.84	2197.60	3.29	241.05	2.55
9	150	2.00	1.50	89.49	6.52	194.97	3839.27	3.12	238.40	4.25
10	180	4.00	4.50	64.16	26.67	220.84	7358.96	3.92	248.70	7.40
11	120	4.00	1.50	45.71	7.80	242.05	1130.47	3.47	290.06	1.40
12	150	6.00	1.50	67.21	6.32	191.60	4289.67	3.10	251.76	4.75
13	180	6.00	3.00	46.82	18.32	214.78	6424.62	3.65	278.85	6.75
14	180	4.00	1.50	79.86	7.15	230.96	8426.15	4.18	265.97	8.30
15	120	6.00	3.00	50.25	17.98	251.00	3299.35	4.16	231.56	3.55

^*^MW, molecular weight; **PDI, polydispersity index

**Table 3:** Analysis of variance of the regression model derived from Box-Behnken design

Source	*d*_f_	*F*-value	*p*-value	
Model	9	23.14	**0.0015**	Significant
X1: temperature	1	31.17	**0.0025**	
X2: sugarcane bagasse:solvent	1	83.15	**0.0003**	
X3: catalyst content	1	2.87	0.1511	
X1 × X2	1	11.57	**0.0187**	
X1 × X3	1	5.06	0.0743	
X2 × X3	1	0.8620	0.3958	
X1^2^	1	64.63	**0.0005**	
X2^2^	1	2.31	0.1888	
X3^2^	1	12.13	**0.0176**	
Lack of fit	3	2.96	0.2624	Not significant

Several studies have highlighted the complexity associated with modelling physicochemical properties of biomass-derived polyols, largely due to the intrinsic heterogeneity of lignocellulosic structures and the simultaneous depolymerization–recondensation reactions occurring during solvothermal liquefaction (Segers et al. 2024). This variability helps explain why, in the present work, RSM provided an excellent predictive model for yield but failed to generate statistically adequate models for *I*_OH_, *A*_n_, *M*_w_ and viscosity—even though such parameters are essential for the industrial applicability of bio-polyols. Comparable difficulties have been reported in other liquefaction studies, prompting the integration of advanced computational tools such as machine learning–assisted RSM to improve predictability (Wu et al. 2024). Furthermore, the behavior observed for catalyst concentration aligns with recent reviews showing that both insufficient and excessive acid levels can negatively affect liquefaction by shifting the balance between depolymerization and secondary repolymerization phenomena (Bontaş et al. 2023; Biswas et al. 2024). The relevance of solvent–biomass interactions noted here is consistent with broader biomass valorization literature, where solvent polarity and chain length have been shown to strongly influence cellulose and hemicellulose solvolysis (Tran and Lee 2023). Additionally, literature supports that the narrowed OH stretching bands and relatively conserved functional group profiles observed in FTIR spectra in biomass liquefaction analyses are related to increased chemical uniformity of –OH groups following solvolytic cleavage reactions (Vale et al. 2019). These connections to contemporary research reinforce the robustness of the present study, while also highlighting emerging opportunities such as microwave-assisted liquefaction, mixed-solvent systems, and catalyst-efficiency enhancement to further improve energy efficiency, reduce acid consumption, and tailor polyol quality (Bontaş et al. 2023; Ingle et al. 2025).

Although yield is considered a key factor and is equally important for making the process industrially feasible, the physicochemical properties are also crucial to obtain bio-polyols with suitable characteristics for polyurethane production. The physicochemical properties exhibit substantial variability due to the inherent heterogeneity of lignocellulosic feedstock. Such variability arises from differences in lignin, cellulose, and hemicellulose ratios across biomass components, as well as chemical distinctions between fractions. Therefore, studies using RSM on biomass-derived polyols have focused on optimizing yield (Wu et al. 2024; Çolakoğlu et al. 2024).

Equation 6 shows the quantitative effect of reaction conditions (*X*_1_, *X*_2_, *X*_3_; temperature, sugarcane bagasse content, and catalyst content, respectively) and the interactions on the bio-polyol yield (Y). This equation indicates factors on the response value.

6 $$Y=-586.81+7.54242{X}_{1}+21.58375{X}_{2}+40.25000{X}_{3}-0.130625{X}_{1}{X}_{2}-0.114278{X}_{2}{X}_{3}-0.707500{X}_{1}{X}_{3}-0.021254{X}_{1}^{2}-0.904687{X}_{2}^{2}-3.68389{X}_{3}^{2}$$

According to Table 3, the *p*-value (0.2624) for the lack of fit was non-significant (*p* > 0.05), indicating that there is no significant lack of fit and that the regression model adequately describes the experimental data. In addition, the high coefficient of determination (*R*^2^ = 0.9766) and adjusted *R*^2^ value (0.9343) further confirm the relevance and good predictive capability of the model.

As shown in Table 3, the temperature and sugarcane bagasse content had significant effect on the yield (*p* < 0.05), while catalyst content was not significant (*p* > 0.05). Moreover, the interaction between temperature and sugarcane bagasse had a significant effect on the yield (*p* < 0.05), whereas the interaction between temperature and catalyst content, as well as sugarcane bagasse content and catalyst content, was not significant (*p* > 0.05).

The bagasse-to-solvent ratio significantly influenced liquefaction yield, consistent with literature reports (Li et al. 2015). El-barbary and Shukry (2008) reported high liquefaction efficiency for agricultural crop residues at solvent-to-biomass ratios ≥ 1:5. However, different solvent ratios showed distinct optimal conditions depending on the biomass type. These differences arise from the heterogeneous lignocellulosic structure of various feedstock (Li et al. 2015).

Temperature is a critical factor in acid-catalyzed liquefaction of lignocellulosic biomass. Previous studies have reported that liquefaction efficiency increases significantly between 130 and 150 °C, with only small improvements at higher temperatures (Hu et al. 2014). In our study, temperature had a significant linear (*p* = 0.0025) and quadratic effect (*p* = 0.0005(. These results indicate that moderate heating supports efficient liquefaction, whereas excessive temperatures can promote recondensation reactions.

Catalyst content did not show a statistically significant linear effect on liquefaction yield (*p* = 0.1511). In contrast, the quadratic term (*X*_3_^2^, *p* = 0.0176) was significant, indicating a non-linear influence in which both insufficient and excessive catalyst levels adversely affect the process. The experimental observation of maximum yield at 3% sulphuric acid is consistent with what has been reported in the literature, where this catalyst level achieves an optimal balance between enhancing liquefaction efficiency and minimizing undesirable recondensation reactions (Li et al. 2015; Wang and Tuohedi 2020).

### Model validation

Based on Eq. 6, the predicted optimal conditions correspond to a temperature of 160.11 °C, a sugarcane bagasse content of 2.00 g and a catalyst concentration of 3.13%, as summarized in Table 4. The validity of this optimal point was confirmed by performing experiments in triplicate under the same conditions. As shown in Table 4, the model predicted a liquefaction yield of 100%, while the experimentally obtained yield was 95.6%, resulting in a low relative error of 4.40%. This close agreement between predicted and experimental results confirms the reliability of the proposed model and demonstrates its applicability for optimizing liquefaction conditions to achieve high bio-polyol yields.

**Table 4:** Comparison of the predicted and observed yield values under various liquefaction conditions

No	Temperature (°C)	Sugarcane bagasse (g)	Catalyst (%)	Predicted yield (%)	Observed yield (%)	Error (%)
1	160.11	2.00	3.13	100	95.60	4.40

### Bio-polyol products and characteristics

In this study, the primary objective during process optimization was to maximize the yield. Following this, the functional suitability of the resulting bio-polyols for polyurethane foam production was assessed. Polyols derived from sugarcane bagasse liquefaction were evaluated through *I*_OH_, *A*_n_, viscosity, *M*_W_, and functionality.

The results presented in Table 2 indicate significant variability in the yield (45–95%) of bio-polyols. Under identical reaction conditions (180 °C, 3 wt.% catalyst), the 94.91% yield was achieved with 2 wt.% bagasse content (high solvent-to-biomass ratio), while 46.82% yield was obtained with 6 wt.% bagasse content (low solvent-to-biomass ratio). This decline in efficiency is consistent with literature findings that agricultural crop residues like bagasse generally require a minimum solvent-to-biomass weight ratio of 5:1 to obtain comparable liquefaction efficiency (Hu et al. 2014). In line with our findings, Wang and Tuohedi (2020) explored the effect of liquefaction conditions on bio-polyol production using cotton stalks as a lignocellulosic source. They found that by increasing the organic solvent: biomass ratio and raising the reaction temperature (from 120 to 160 °C), the polyol yield improved and the residual solid component decreased.

The *I*_OH_ also ranged between 182.4 (150 °C, 4.5% catalyst, and 6 g of bagasse) and 251 mg KOH/g (120 °C, 3% catalyst, and 6 g of bagasse). These data imply a potential decline in OH functionality with increased catalyst concentration at elevated temperatures. Literature confirms that reaction temperature and catalyst content strongly influence this parameter (Hu et al. 2014; Wang et al. 2020; Erdem et al. 2023). This *I*_OH_ reduction is probably due to the dehydration and thermal-oxidative degradation of the liquefaction solvent, as well as interactions between the solvent and the dissolved liquefied substances (El-barbary and Shukry et al. 2008). However, the *I*_OH_ obtained in this study were in the same range with those obtained during the liquefaction of agricultural crop residues studied (Li et al. 2015). Furthermore, our results are consistent with those reported by Vale et al. (2019), who prepared bio-polyol from cork and eucalyptus powder to synthesize PU foam. They employed a combination of diethylene glycol and 2-ethyl hexanol as organic solvents with p-toluene sulfonic acid as catalyst, achieving a bio-polyol yield of 79%. The *I*_OH_ for the cork powder bio-polyol was 272 mg KOH g^−1^, while for the eucalyptus bio-polyol, it was 197 mg KOH g^−1^. The *I*_OH_ of the samples 3, 4, and 9 were between the typical ranges reported for rigid PUs (250–1000 mg KOH/g) and flexible PUs (28–160 mg KOH/g) (Hernández-Ramos et al. 2023). Given their intermediate values (∼ 195 to ∼ 233 mg KOH/g), they are closer to the characteristics associated with elastic polyurethane manufacture.

With regard to the *A*_n_, it ranged from 6.32 to 27.11 mg KOH/g. The increase in *A*_n_ can be explained by the formation of organic acids from the decomposition of lignocellulosic biomass and/or the oxidation of polyhydric solvents (Li et al. 2015; Jasiūnas et al. 2020), exacerbated by the strong oxidizability of high sulphuric acid concentrations (> 4.5%) promoting secondary reactions (Wang et al. 2007). As a result, the need for complete neutralization or excessive use of bases such as NaOH or MgO in the formulation may be decreased (Amran et al. 2019). Neutralized polyols can be utilized either by themselves or as partial replacements for petroleum-based polyols in the production of PU foams (Li et al. 2015). The *A*_n_ measured in this study (6.32 and 27.11 mg KOH/g) correspond closely with values reported in the literature (Erdem et al. 2023; Li et al. 2015; Hu et al. 2014).

*M*_W_ analysis reveals a wide distribution among the liquefied samples, ranging from 1130.47 to 22,926.36 g/mol, with PDI values between 1.40 and 18.40. Higher *M*_W_ values are consistently accompanied by higher PDI values, reflecting the dynamic balance between decomposition and recondensation processes. *M*_W_ of polyols typically increases during liquefaction as a result of the release of macromolecular fragments from biomass. As the reaction progresses, these fragments gradually degrade, leading to a reduction in *M*_w_ and a narrower *M*_W_ distribution. However, when pronounced recondensation reactions occur, *M*_w_ may increase again (Hu et al. 2014; Yan et al. 2008). Additionally, the rise in *M*_w_ results from re-polymerization reactions, which are promoted by the presence of an acid catalyst (Lee et al. 2016). The *M*_W_ values of bio-polyols (samples 3, 4, 9) are within the range of 2000–10,000 g/mol, which is suitable for elastic PU manufacture (Ionescu et al. 2012). Their PDI values are between 4 and 8.

To better understand the evolution of the physicochemical properties observed during liquefaction, a plausible reaction mechanism for the conversion of sugarcane bagasse under the employed solvothermal conditions is proposed. Initially, the sulphuric acid catalyst promotes the hydrolysis of glycosidic bonds present in cellulose and hemicellulose, as well as the cleavage of ether linkages within lignin, generating soluble oligomers and low-molecular-weight reactive intermediates (Wang and Chen 2007; Zhang et al. 2020). These depolymerization reactions facilitate the disruption of the lignocellulosic matrix and the transfer of biomass-derived fragments into the liquid phase. Simultaneously, PEG 400 acts not only as a solvent but also as a reactive polyhydric alcohol capable of stabilizing the generated intermediates through alcoholysis and etherification reactions, thereby limiting the formation of insoluble residues and enhancing liquefaction efficiency (Lee and Lee 2016; Olszewski et al. 2023). As the reaction progresses, hydroxyl-containing degradation products from cellulose, hemicellulose and lignin can become incorporated into PEG-derived structures, leading to the formation of bio-polyols with increased hydroxyl functionality and improved suitability for polyurethane synthesis (Hu et al. 2014; Lee and Lee 2016). However, under severe reaction conditions, such as elevated temperatures or excessive acid concentrations, secondary condensation and repolymerization reactions may occur, producing larger molecular structures and broader molecular-weight distributions (Hu et al. 2014; Zhang et al. 2020). This interpretation is consistent with the increase in molecular weight and PDI observed in some of the liquefied samples and supports the hypothesis that the final bio-polyol properties result from a dynamic balance between depolymerization, PEG-assisted solvolysis, stabilization of reactive intermediates, and recondensation reactions.

The functionality values are defined as the average number of OH groups per molecule. For elastic PUs, the required functionality is typically between 2 and 3. Relatively low values lead to a reduced crosslink density, which in turn permits higher mobility of the polymer chains (Ionescu et al. 2012). Samples 3, 4, and 9 exhibited functionalities in the range of 3 to 4. Nevertheless, based on their *I*_OH_ and *M*_w_ values, they can still be regarded as appropriate for elastic PU manufacture.

The apparent viscosity values of the polyol dispersions obtained in this study ranged from 187.56 to 469.03 mPa · s, which are lower than those reported in the literature (Hu et al. 2014). In our work, PEG 400 was employed as the liquefaction solvent, consistent with Olszewski et al. (2023), who observed comparable trends in hydroxyl number and viscosity. Moreover, Luo et al. (2013) demonstrated that PEG-based bio-polyols generally exhibit lower viscosities compared to those synthesized with other solvents, such as glycerol. Hu et al. (2014) documented that bio-polyols with viscosities ranging from 300 to 45,000 mPa · s are typically associated with the production of rigid and semi-rigid polyurethane foams. Highly viscous polyols reduce mixing efficiency and adversely impact polymerization in polyurethane foams (Erdem et al. 2021). The viscosity range observed is moderate, suggesting the bio-polyols could be readily processed without severe flow issues, favorable for scalable elastic PU production.

In this study, it was crucial to focus on the reaction conditions under which the highest yield of bio-polyol, at the same time, the best qualitative characteristics could be attained. If the reaction conditions are not optimized based on highest achievable bio-polyol yield, a significant portion of the biomass may remain unliquefied or in solid form. Therefore, it can be concluded that reaction conditions are more critical for bio-polyol yield than its qualitative parameters. Out of the total 15 runs, three high-yield samples were identified, which also suggested possible applications in elastic foam. FTIR spectra were obtained for these selected samples (3, 4, and 9) and compared with the spectrum of plain sugarcane bagasse prior to liquefaction.

### Effects of liquefaction conditions on functional groups

**Fig. 2:**
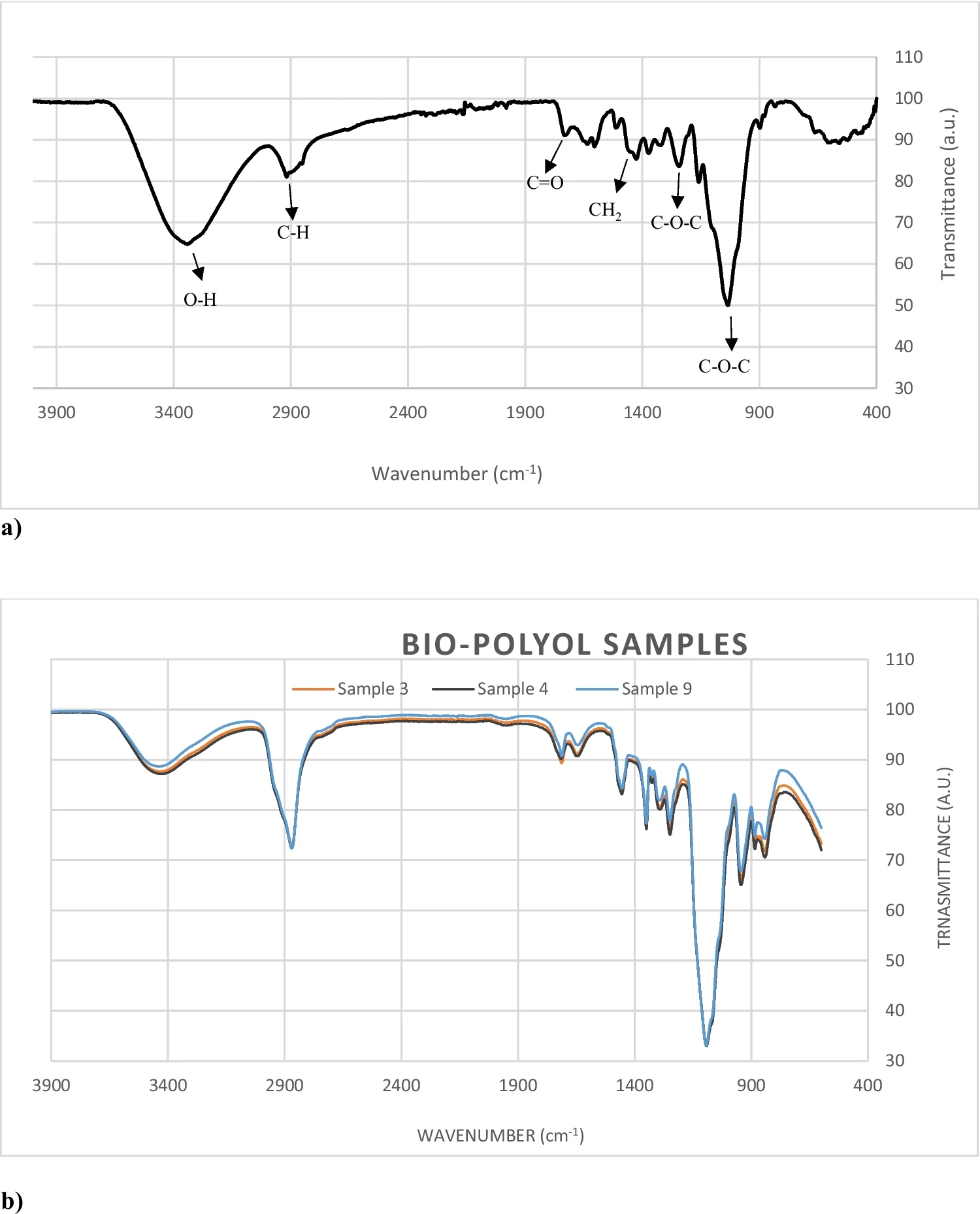
FTIR spectra comparison of **a** plain sugarcane bagasse and **b** sugarcane bagasse liquefied under various conditions. Production conditions for samples 3, 4, and 9 (highest yield) are provided in Table 2

According to FTIR analysis (Fig. 2a), the plain sugarcane bagasse exhibited broad absorption bands at 3332 cm^−1^ and 2897 cm^−1^ which could be corresponded to the OH group and C-H stretching in methyl and methylene groups, respectively (Hamin et al. 2023). Moreover, the peak at 1731 cm^−1^ is indicative of the carbonyl functional group of the hemicellulose and lignin (Ban et al. 2022). The absorption band at 1422 cm^−1^ is also associated with the CH_2_ scissoring motion in cellulose (El Naeem et al. 2022). Furthermore, the absorption band at 1032.50 cm^−1^ is attributed to the C-O-C pyranose ring skeletal vibration in cellulose (Ban et al. 2022). By comparing the FTIR spectra of plain sugarcane bagasse with the bagasse liquefied under various conditions (Fig. 2b), it can be observed that the OH band of the plain bagasse was broader and more intense than the OH bands of the liquefied bagasse samples. In contrast, the CH bands of the latter samples were more distinct compared to the plain bagasse.

Other functional groups of the liquefied bagasse samples display slight differences when compared to plain bagasse. These results indicate that the liquefaction process primarily affects the OH and CH groups within the biomass. The synthesis of bio-polyol from sugarcane bagasse involves significant chemical transformations, particularly in the –OH and methyl/methylene (–CH) functional groups. Sugarcane bagasse primarily contains cellulose, hemicellulose, and lignin, with abundant –OH groups attached to saccharide units and –CH groups present in aliphatic chains and lignin’s methoxy groups. During the bio-polyol synthesis process, which typically employs liquefaction or chemical modification methods, the structure of the raw biomass is disrupted, leading to an increase in free and accessible OH groups. This is evidenced by enhanced –OH stretching bands in spectroscopic analyses, indicating a higher *I*_OH_ that directly contributes to improved reactivity for subsequent polyurethane production (Xie et al. 2015).

The observed narrowing of the OH stretching band in FTIR spectra after converting lignocellulosic biomass into polyols indicates a significant reduction in hydrogen bonding complexity and an increase in the chemical uniformity of OH groups. In untreated biomass, the broad OH absorption band ranging from 3200 to 3600 cm^−1^ results from a heterogeneous distribution of OH functionalities in cellulose, hemicellulose, and lignin (Gurgel et al. 2021). These groups extensively participate in intra- and intermolecular hydrogen bonding, which causes spectral broadening. During chemical processes like acid liquefaction, solvolytic reactions break down the biomass matrix and introduce more reactive and soluble OH groups with less bonding diversity. This transformation leads to a more distinct OH stretching band in the resulting polyol. Furthermore, chemical modifications add aliphatic OH groups with reduced hydrogen bonding capacity, further sharpening the FTIR spectral features. These results highlight FTIR spectroscopy’s usefulness as a tool for monitoring the structural changes and functional group simplification during biomass conversion into polyols (Vale et al. 2019).

Conversely, the changes in –CH groups reflect the complex degradation and reorganization of the carbohydrate and lignin frameworks. Some aliphatic –CH groups may be lost or formed depending on side reactions such as demethylation, ring-opening, or the incorporation of additional polyhydric alcohols like glycerol. This interplay between the –OH and –CH groups is pivotal for tailoring bio-polyol properties. Increased –OH availability enhances crosslinking potential in polyurethanes, while modifications in –CH content influence the polyol’s structural characteristics and mechanical performance. Therefore, understanding and controlling these functional group changes is essential for optimizing bio-polyol synthesis from sugarcane bagasse, contributing to the development of sustainable polyurethane materials (Tran et al. 2022). The FTIR spectra of bio-polyols obtained under different liquefaction conditions exhibited highly similar profiles, indicating that the chemical structures of the products remain consistent despite variations in reaction parameters. This spectral similarity confirms the preservation of key functional groups.

### Conclusions

The results demonstrate that sugarcane bagasse is a promising and sustainable feedstock for the production of bio-polyols through acid-catalyzed liquefaction using PEG400. Process optimization by response surface methodology enabled the identification of temperature and biomass loading as the most influential variables governing liquefaction yield, while the developed model exhibited high predictive accuracy and experimental reliability. The bio-polyols obtained under optimized conditions exhibited physicochemical properties, including hydroxyl number, molecular weight, functionality, viscosity, and PDI, within ranges reported for polyols used in elastic polyurethane systems. Although polyurethane formulations were not prepared in the present work, these findings suggest their potential suitability for such applications. Future studies should focus on the synthesis, characterization, and performance evaluation of polyurethane materials derived from these bio-polyols to experimentally validate their application potential. This work highlights an effective strategy for the valorization of agricultural residues and contributes to the advancement of more sustainable, resource-efficient, and circular polyurethane production systems. Future studies should address the recovery and recycling of PEG 400 to assess the economic feasibility and environmental performance of the proposed liquefaction process at larger scales.

## Data Availability

The datasets used and/or analyzed during the current study are available from the corresponding author on reasonable request.
